# A single‐centre study evaluating a geriatric screening tool in oncology phase I trial patients

**DOI:** 10.1002/cnr2.2083

**Published:** 2024-06-25

**Authors:** Mary Van Zyl, Anne Barell, Bridget Cooley, Janet Hanwell, Josie Parlak, Udai Banerji, Johann De Bono, Adam Sharp, Juanita Lopez, Nicolo Matteo Luca Battisti, Anna Minchom

**Affiliations:** ^1^ Drug Development Unit Royal Marsden Hospital/Institute of Cancer Research Sutton UK; ^2^ Senior Adult Oncology Programme Royal Marsden Hospital Sutton UK; ^3^ Breast Unit & Senior Adult Oncology Programme Royal Marsden Hospital Sutton UK

**Keywords:** clinical trial, geriatric assessment, phase I

## Abstract

**Background:**

Though cancer is more prevalent in the older population, this patient group are underrepresented in phase I oncology trials.

**Aims:**

We evaluated the use of a geriatric screening tool (SAOP3) in patients of 70 years of age or older who attended a Phase I Clinical Trials Unit, with the aim of assessing the feasibility of the tool and identifying potential unmet needs in this patient group.

**Methods:**

Twenty‐two patients over the age of 70 completed the SAOP3 questionnaire. Geriatric impairments and needs were analysed with descriptive statistics. Qualitative responses were grouped in themes using structured thematic analysis.

**Results:**

All of patients triggered at least 1 geriatric domain, most commonly mobility. Six core themes were identified as being important to the patient including family, friends and positivity. On cognition assessment over 20% of patients triggered as requiring further cognitive assessment. The group had a relatively high screen fail risk.

**Conclusion:**

In conclusion, routine geriatric screening withSAOP3 was feasible and identified areas of patient need. Results highlight the prevalence of psychological distress and cognitive impairment. Geriatric screening offers an opportunity for prehabilitation prior to trial and support during trial participation to optimise safety and improve trial access.

## 1 BACKGROUND

Phase I trials in Oncology are essential to drive drug development forward and offer patients the opportunity to access innovative experimental options when standard treatments have been exhausted. Phase I oncology trials are time intensive and yield unknown risks of toxicity and chances of meaningful benefit of approximately 10%–20% based on biomarker sensitivity.^1^ The selection process is rigorous, with strict eligibility criteria to be met. Nonetheless, about 20% of patients withdraw early from a phase I trial^2^: this is undesirable both for the patient wellbeing and trial efficiency.

Cancer is a disease of ageing, with individuals aged ≥65 years representing 54% of cancer incidence and 70% of cancer mortality.^3^ Nonetheless, older patients are under‐represented in phase I trials.^4^ Their underrepresentation drives inequalities and means data derived from early phase trials, to support future drug development, is derived in populations not representative of those seen in routine clinical practice.^5^ Toxicity, dosing, scheduling, concurrent medications, predictive symptoms, biomarkers and laboratory findings may be significantly different in older individuals compared to younger patients, thus recruiting only the younger populations may lead to inaccurate assessment of drug tolerance in the population as a whole. Previous data did not identify age as predictive factor for early trial withdrawal suggesting older patients can be safely recruited to early phase trials.^2^ Given that the recommended phase II dose and pharmacokinetics are established in the phase I trials, it is important to include an older population in this early phase of drug testing by means of facilitating their recruitment and retention on phase I trials.

Substantial evidence and international guidelines support the routine use of geriatric assessments to inform treatment decisions in older adults with cancer.^6^, ^7^, ^8^ Comprehensive geriatric assessment (CGA) involves the systematic evaluation of key domains relevant to the well‐being of older adults (comorbidities, functional status, cognitive status, nutritional status, concurrent medications, mood, social support and activity) and personalised geriatric assessment (GA)‐driven interventions.^9^ GA‐driven interventions are multi‐modal including tailored physiotherapy, occupational therapy, nutritional counselling, psychological support, advice on access to financial aid, medication review and optimisation of co‐morbid medical conditions. Older patients warranting CGA may be identified with geriatric screening tools, that are feasible to implement also in busy oncology practices.^7^ Nonetheless, the role of geriatric assessments is yet to be explored in the context of early phase I trials as inclusion or end‐point assessments. Previous literature searches have identified few examples of geriatric screening tools being utilised in phase I trial endpoints and, where included, tend to be poorly characterised or unvalidated and therefore limited in their conclusions.^10^, ^11^ We hypothesise that a geriatric screening tool is feasible in a Phase I trials unit and ultimately allow early identification of unmet needs of this population to be identified, thus helping patients be recruited more often to Phase I trials.

The SAOP3 is a validated, patient‐reported, short screening questionnaire aimed at identifying patients requiring CGA and is currently in use in our Institution for patients aged ≥70 years being considered for systemic anticancer therapy.^12^ Validation data is reported and updated from the prior SAOP2 tool.^13^ We chose SAOP3 since this is validated in various oncology settings and since this has been adopted across various tumour‐specific Units in our Institution.

We evaluated the feasibility of geriatric screening and the needs of older patients requiring additional interventions using a geriatric screening tool, SAOP3, at a UK‐based dedicated Phase I Clinical trials unit.

## 2 METHODS

Patients aged 70 years and older seen in our Drug Development Unit (DDU) and assessed at that visit as suitable for trial entry were eligible to participate between August 2022 and November 2022.

Patients were approached by the DDU team to offer a geriatric screening using the SAOP3 tool. All patients over the age of 70 were approached in person to participate at the time they attended their new patient appointment in the DDU. The SAOP3 includes questions on functional status (activities of daily living), nutrition, mood, speech and language, polypharmacy, social support, self‐reported health and quality of life and includes a cognitive screening test (Mini‐Cog) and a “What matters to you?” question.^12^ This was administered in person at the time of the patient's first assessment, the questionnaire portion was completed independently by the patient and the Mini‐cog element was administered by the nurse.

Feasibility was assessed by the number/percentage of patients completing the SAOP3 tool and number/percentage of complete responses received. In order to describe the additional needs of the older patient using the SAOP3, individual domains triggering multidisciplinary referrals and interventions were recorded. Geriatric impairments and needs were analysed with descriptive statistics.

Answers to the “What matters to you?” question were also recorded to provide insight on patient care goals alongside individual needs. Two investigators (MVZ, AM) reviewed the answers to the “What matters to you?” question and conducted a structured thematic analysis to group them in overarching themes. No answers were excluded from this analysis and the themes were ranked in order of frequency.

## 3 RESULTS

Twenty‐two patients were approached. All patients approached to participate completed the questionnaire in full with no missing information. No patients refused to participate. No concerns were raised by patients completing the SAOP3 questionnaires or by the clinician or nurse collecting them.

Baseline characteristics of the study population are outlined in Table 1. The age range was 70–78 years (median 73.5 years). The cohort included 10 female and 12 male patients. A number of malignancies were represented. The Eastern Cooperative Oncology Group (ECOG)^14^ performance status documented by a DDU clinician was 0 in 3 patients, 1 in 16 patients, 2 in 2 patients and not documented in 1 patient.

**TABLE 1:** Characteristics of study participants.

Age	70–78 years (median 73.5 years)
Male: Female	10:12
ECOG Performance Status	PS0:3, PS1:16, PS2:2, 1: not documented
Tumour Type	Metastatic castrate resistance prostate cancer: 2 Mesothelioma: 1 Breast cancer: 2 Glioblastoma: 2 Melanoma: 3 Lung cancer: 4 Endometrial cancer: 1 Pancreatic cancer: 3 Colorectal cancer: 1 Sarcoma: 1 Hodgkin lymphoma: 1 Oesophageal cancer: 1 Ovarian cancer: 1

Abbreviation: ECOG: Eastern Cooperative Oncology Group.

**FIGURE 1:**
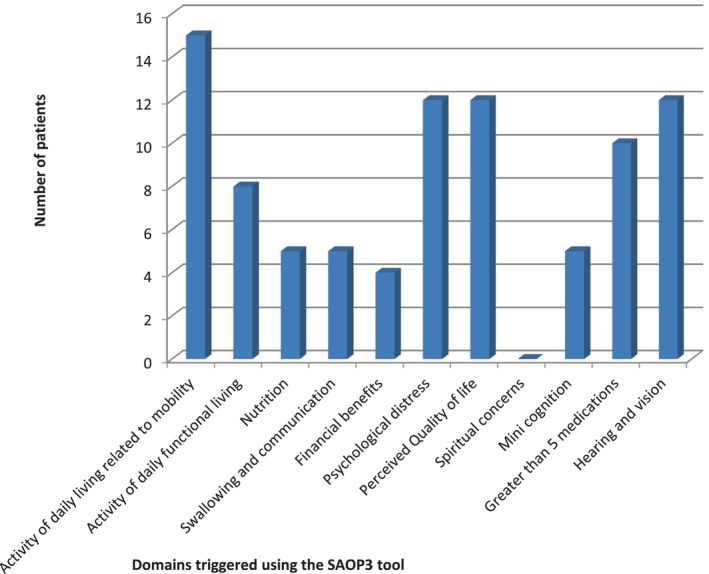
Number of patients with specific needs based on SAOP3.

**FIGURE 2:**
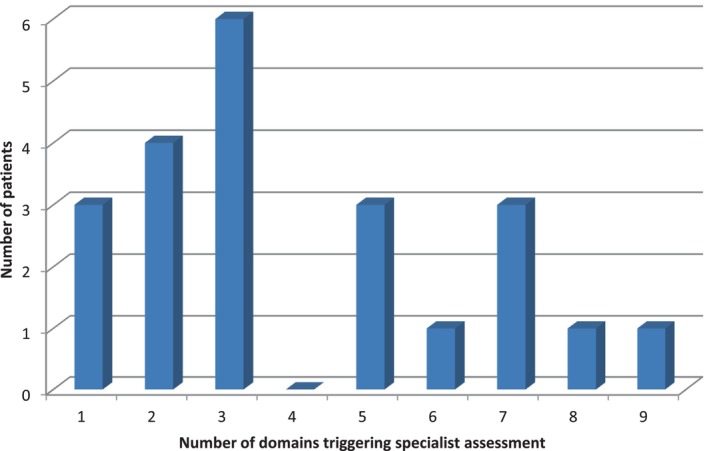
Number of needs for each individual patient completing SAOP3.

All patients triggered for at least 1 geriatric domain on SAOP3 warranting further assessments and interventions. Figure 1 outlines the range of needs across all domains. The most common need pertained to mobility in 15 out of 22 and warranted a referral to physiotherapy/occupational therapy referral. Patients triggered for a median of 4 domains (range 1–9). Figure 2 shows the number of needs for each individual patient.

The following six core themes were identified following a systematic thematic analysis. The most commonly repeated comments related to the importance of personal relationships. The number of times each theme was identified is in brackets below:

1. Family and friends: being with, looking after, worrying about (“I want to be a good mother”) (15)
2. Positivity: not giving up, beating cancer, getting treatment, hope and faith (“Faith‐hopeless optimism‐ Beating this cancer”) (5)
3. Quality and quantity of life (“To live well for as long as she can, enjoy time left.”) (4)
4. Suicidal or negative thoughts (“I have thought about suicide and how I would do it”) (1)
5. Occupation (“My career in the RAF”) (1)
6. Pets (“My dogs”) (1)

Of the 22 patients assessed, 4 patients were subsequently not deemed fit for phase 1 studies following the initial visit and 5 patient were allocated to a clinical trial but could not proceed with recruitment for reasons of fitness or blood parameters (a trial “screen fail”); thus highlighting the risk of clinical deterioration in this patient group. 7 patients were recruited successfully in a trial. The remaining 6 patients did not enter a trial for reasons not related to their fitness: 2 did not have progressive disease; 3 patients opted for a standard treatment option, and 1 patient did not have measurable disease based on RECIST criteria.

On the Mini‐Cog, 5 patients triggered for further cognitive assessment as per the SAOP scoring criteria. Of the 5 patients warranting further assessments on Mini‐Cog, one screen failed, 3 were not allocated to a trial and one was recruited in a trial.

Nine patients were not recruited in phase I trials owing to fitness or abnormal blood parameters. For these patients, the reasons for non‐entry to trial and domains triggered were reviewed. In three patients the reason for non‐trial entry could be potentially a cause of the domains triggered; a patient who screen failed due to peripheral neuropathy impacting on 6 SAOP3 domains, a patient with a decrease in Performance Status (PS) due to cancer progression with 5 SAOP3 domains triggered and a patient with worsening pleural effusion triggering on one SAOP3 domain. However, for most patients (6 out of 9), the reasons for non‐entry to trial were asymptomatic test abnormalities (e.g., lipase rise or occult cardiovascular disease on ECG). Such exclusion criteria are commonly mandated in phase I trial protocols (rather than being specific to individual phase I trials).

We also compared these 9 patients with those who successfully entered trial. For those who did not start trial (*n* = 9), patients triggered on an average on 4.4 domains (range 1–7). For those who successfully started trial (*n* = 7), patients triggered on an average of 3.4 domains (range 2–6).

## 4 DISCUSSION

To our knowledge, this study is the first analysis documenting the feasibility and outcomes of geriatric screening for patients aged ≥70 years reviewed in a phase 1 trial unit in a large cancer centre. This is key to ensure equal access to early phase trials for older adults with cancer and to adequately support them while they undergo experimental treatments. In this study the most reported needs related to mobility among additional needs also identified. Previously there have been attempts to include geriatric screening tools as baseline assessments, inclusion criteria and outcome measures in oncology trials, albeit with a lack of a consensus approach and rarely within phase I trials.^10^, ^11^ However, we report the potential for the SAOP3 tool in patients prior to trial participation aiming to provide holistic and prehabiliation care, ultimately to ease compliance and promote a positive experience for phase I trial participants.

Routine geriatric screening with SAOP3 was feasible with universal compliance in our new patient phase I clinic. Importantly, no patients declined to complete SAOP3 screening, with no missing data or concerns with completion. The high rates of patient acceptability are consistent with findings from patients completing SAOP3 who are receiving standard anticancer treatments.^7^

Patient referrals to the DDU new patient clinic are typically screened ahead of their first consultation based on existing documentation available on their referral letter; including performance status, co‐morbidities and laboratory results to determine broad phase 1 trial eligibility. Therefore, patients completing SAOP3 geriatric screening had been judged suitable for phase I trial assessment.

Nonetheless, we have documented high levels of additional needs in this population, with all patients triggering for ≥1 geriatric domain on SAOP3.

Geriatric screening offers a unique opportunity to refer patients to a dedicated geriatric oncology or geriatrics team in order to develop a personalised programme of prehabilitation prior to and support during phase I trials. The Senior Adult Oncology Programme was developed at our Institution to take referrals for patients with needs documented on SAOP3 screening and address them with interventions including pharmacy review for polypharmacy (e.g., deprescribing), physiotherapy and occupational therapy input for mobility issues (e.g., balance training, fall prevention discussion, home exercise programme), dietetics input for nutritional problems (e.g., oral nutritional supplements, assistance with meal preparation, oral care input), psychological medicine input for psychological distress and others. To improve rates of trial allocation speedy review and management of patients in the short period between being seen in new patient clinic and commencement of trial (typically 2–3 weeks) may represent some challenges. The cost implications and implementation of such a service are currently being assessed in ongoing work in our Institution.^12^

We observed a relatively high rate of deterioration resulting in trial ineligibility prior to consenting (18.2%, 4/22) and high screen fail risk (22.7%, 5/22) in this group, highlighting a key level of unmet need. Therefore, this analysis highlights the need for prospective evaluation of the effect of geriatric screening on phase 1 trial screening, eligibility and retention.

In comparison of those who were not included in phase I trials with those who entered trial there was little difference in the number of domains triggered on SAOP3 and it also appeared that a significant number of patients screen failed for reasons unrelated to the domains triggered on SAOP3 which would suggest that implementation of SAOP3 and prehabilitation may have limited impact on patient recruitment. This analysis is limited by the small sample size of these subgroups and will be further assessed on role out of the SAOP3 in standard practice.

In addition, our analysis highlights the prevalence of psychological distress in older adults with cancer being considered for phase 1 trials. This patient group faces unique psychosocial stressors, being older and affected by an end‐stage cancer albeit not actively dying. Wider awareness of the prevalence of these stressors and interventions may help clinicians and support teams to address their needs more effectively. For example, regularly asking older adults about their feelings of dependency, psychological distress and dynamic within their family as traditional roles shift over time, allows clinicians to address key concerns for this specific patient population.^13^

The qualitative data obtained, highlighted that more patients commented on the importance of personal relationships than anything else. It would be plausible to assume priorities shift during one's life span, identifying that older patients may have different priorities than a younger cohort. An awareness of individual's personal priorities will help guide discussions with patients about the choice of trial participation, allowing for improved informed consent.

A triggered domain in cognitive screening does not imply a diagnosis of cognitive impairment and may be biased in a busy outpatient oncology clinic environment. Nonetheless, notably we documented that 20% of patients required further cognitive evaluation. Cognitive impairments may benefit from specialised interventions aiming to produce a meaningful impact on quality of life. Cognition is also critical to support the informed consent procedure, compliance with drug dosing instructions and recall of specific symptomology. These considerations are key to the safe delivery of phase I trials. Increased awareness of its importance is vital to support this vulnerable patient group.

This study has some limitations. These include a relatively small sample size and the fact that it was conducted in a single centre with a single geriatric screening tool. Although the SAOP3 tool has high predictive value for identifying needs,^10^ further work to assess its applicability beyond one centre is warranted; as well as prospective data collection to map and quantify the health care resource implications of implementation and impact on trial recruitment, screen fail and patient retention on trials. Although a positive impact of CGA on patient outcomes including unplanned hospitalisations, severe toxicities and quality of life on systemic anticancer therapy has been demonstrated in this population,^15^, ^16^, ^17^ its impact on outcomes for older patients enrolled in phase I trials is yet to be demonstrated and the lack of such data is a limitation of our analysis. Also, in our analysis we have not evaluated the impact of SAOP3 geriatric screening on patients' prognosis because this geriatric screening tool was designed to select patients for CGA (rather than for predicting their prognosis). To this purpose, this specific tool was found to be more sensitive in highlighting patients' needs compared with G8, another widely used geriatric screening tool.^12^

Recommending the SAOP3 tool in the oncology clinical trial environment maybe challenging without access to a dedicated geriatric oncology team who can initiate a comprehensive assessment with appropriate interventions. It could be argued however that identifying unmet needs of the older adult would trigger internal referrals. Indeed, clinical teams would have a professional obligation to address patients' needs to offer holistic and patient centred care.

In addition, there is lack of consensus for the age at which geriatric assessments are appropriate, although the American Society of Clinical Oncology recommends geriatric assessments for those aged and older 65 years.^8^, ^18^ The next stage of implementation of the SAOP3 tool should explore other age restraints to ensure optimum benefit versus service costs.

In conclusion, SAOP3 is ideal for use in a phase 1 trial unit outpatient setting as the questionnaire is short and easy to use. Moreover, the SAOP3 tool identifies complex needs in older patients being considered for phase I trials and may offer an opportunity to operationalise geriatric screening in a multidisciplinary setting to tailor care and services, optimise treatment safety and improve access to innovation as a prehabilitation service in this group of patients.

## AUTHOR CONTRIBUTIONS

**Mary van Zyl:** Conceptualization, formal analysis, investigation, methodology, writing original draft, writing review. **Anne Barell:** Conceptualization, methodology. **Bridget Cooley:** Investigation. **Janet Hanwell:** Investigation. **Josie Parlak:** Investigation. **Udai Bernerji:** Resources, supervision, writing review/edit. **Johann de Bono:** Resources, supervision, writing review/edit. **Juanita Lopez:** Resources, supervision, writing review/edit. **Nicolo Matteo Luca:** Conceptualization, software, supervision, writing original draft, writing review/edit. **Anna Minchom:** Conceptualization, supervision, resources, writing original draft, writing review/edit, validation.

## FUNDING INFORMATION

This study represents independent research supported by the National Institute for Health Research (NIHR) Biomedical Research Centre at the Royal Marsden NHS Foundation Trust and the Institute of Cancer Research. The views expressed are those of the authors and not necessarily those of the NIHR or the Department of Health and Social Care.

## CONFLICT OF INTEREST STATEMENT

Mary Van Zyl, Anne Barell, Bridget Cooley, Janet Hanwell, and Josie Parlak have none to declare. Udai Banerji: Employee of the Institute of Cancer Research which has commercial interests in HSP90, PI3K, AKT, HDAC, BRAF, CHK1, MPS‐1, Molecular glues and HSF‐1 pathway inhibitors Speaker's Bureau for: None Grant/Research support from: Verastem Oncology, Chugai Pharmaceuticals, Carrick Therapeutics, BTG, Avacta Stockholder in: None Honoraria for advisory board from: Pegascy, Carrick Therapeutics, Boehringer Ingelheim, Jannsen. Johann De Bono has served on advisory boards and received fees from companies including Amgen, Astra Zeneca, Astellas, Bayer, Bioxcel Therapeutics, Daiichi, Genentech/Roche, GSK, Harpoon, ImCheck Therapeutics, Janssen, Merck Serono, Merck Sharp & Dohme, Pfizer, Sanofi Aventis. Is an employee of The Institute of Cancer Research, which have received funding or other support for his research work from AZ, Astellas, Bayer, Cellcentric, Daiichi, Genentech, Genmab, GSK, Janssen, Merck Serono, MSD, Menarini/Silicon Biosystems, Orion, Sanofi Aventis, Sierra Oncology, Taiho, Pfizer, Vertex. The ICR has a commercial interest in abiraterone and PARP inhibition in DNA repair defective cancers and PI3K/AKT pathway inhibitors (no personal income). Is named as an inventor, with no financial interest for patent 8,822,438, submitted by Janssen that covers the use of abiraterone acetate with corticosteroids. Adam Sharp Is an employee of the ICR, which has a commercial interest in abiraterone, PARP inhibition in DNA repair defective cancers, and PI3K/AKT pathway inhibitors (no personal income). Adam Sharp has received travel support from Sanofi, Roche‐Genentech and Nurix, and speaker honoraria from Astellas Pharma and Merck Sharp & Dohme. He has served as an advisor to DE Shaw Research and CHARM Therapeutics. Adam Sharp has been the CI/PI of industry‐sponsored clinical trials. Juanita Lopez: Consultant or advisory role for the following pharma companies; Genmab, Novartis, Basilea, Roche/Genetech, CureTeq, Ellipses Pharma & Pierre Fabre. Involved in Grant/Research funding for Roche/Genetech, Basila, Astex pharmaceuticals. Travel expenses covered for Basilea, Roche/Genetech. Nicolo Matteo Luca Battisti: Advisory board: Pfizer, Abbott, Sanofi, Astellas; Travel grants: Exact Sciences, Pfizer, Lilly, Novartis; Speaker fees: Pfizer, AbbVie, Roche, Sanofi, Novartis, Servier, Gilead, AstraZeneca, Lilly. Anna Minchom has served on advisory boards for Janssen Pharmaceuticals, Merck Pharmaceuticals, Takeda Pharmaceuticals and Genmab Pharmaceuticals. Has received honoraria from Chugai Pharmaceuticals, Novartis Oncology, Faron Pharmaceuticals, Bayer Pharmaceuticals, Merck Pharmaceuticals, GSK and Janssen Pharmaceuticals. Has received expenses from Amgen Pharmaceuticals and LOXO Oncology. Has received research funding from Merck Pharmaceuticals and MSD.

## ETHICS STATEMENT

Approval was gained from the local ethics committee.
